# The Transferable Resistome of Produce

**DOI:** 10.1128/mBio.01300-18

**Published:** 2018-11-06

**Authors:** Khald Blau, Antje Bettermann, Sven Jechalke, Eva Fornefeld, Yann Vanrobaeys, Thibault Stalder, Eva M. Top, Kornelia Smalla

**Affiliations:** a Julius Kühn-Institut, Federal Research Centre for Cultivated Plants, Braunschweig, Germany; b Justus Liebig University Giessen, Institute for Phytopathology, Gießen, Germany; c Department of Biological Sciences, University of Idaho, Moscow, Idaho, USA; d Institute for Bioinformatics and Evolutionary Studies, University of Idaho, Moscow, Idaho, USA; CEH-Oxford

**Keywords:** *Escherichia coli*, IncF, IncI, antibiotic resistance, horizontal gene transfer, real-time PCR

## Abstract

Produce is increasingly recognized as a reservoir of human pathogens and transferable antibiotic resistance genes. This study aimed to explore methods to characterize the transferable resistome of bacteria associated with produce. Mixed salad, arugula, and cilantro purchased from supermarkets in Germany were analyzed by means of cultivation- and DNA-based methods. Before and after a nonselective enrichment step, tetracycline (TET)-resistant Escherichia coli were isolated and plasmids conferring TET resistance were captured by exogenous plasmid isolation. TET-resistant E. coli isolates, transconjugants, and total community DNA (TC-DNA) from the microbial fraction detached from leaves or after enrichment were analyzed for the presence of resistance genes, class 1 integrons, and various plasmids by real-time PCR and PCR-Southern blot hybridization. Real-time PCR primers were developed for IncI and IncF plasmids. TET-resistant E. coli isolated from arugula and cilantro carried IncF, IncI1, IncN, IncHI1, IncU, and IncX1 plasmids. Three isolates from cilantro were positive for IncN plasmids and *bla*_CTX-M-1_. From mixed salad and cilantro, IncF, IncI1, and IncP-1β plasmids were captured exogenously. Importantly, whereas direct detection of IncI and IncF plasmids in TC-DNA failed, these plasmids became detectable in DNA extracted from enrichment cultures. This confirms that cultivation-independent DNA-based methods are not always sufficiently sensitive to detect the transferable resistome in the rare microbiome. In summary, this study showed that an impressive diversity of self-transmissible multiple resistance plasmids was detected in bacteria associated with produce that is consumed raw, and exogenous capturing into E. coli suggests that they could transfer to gut bacteria as well.

## Abstract (precis)

Produce is one of the most popular food commodities. Unfortunately, leafy greens can be a reservoir of transferable antibiotic resistance genes. We found that IncF and IncI plasmids were the most prevalent plasmid types in E. coli isolates from produce. This study highlights the importance of the rare microbiome associated with produce as a source of antibiotic resistance genes that might escape cultivation-independent detection, yet may be transferred to human pathogens or commensals.

## INTRODUCTION

Despite its benefit to human health, consumption of produce is increasingly recognized as a source of pathogenic bacteria, antibiotic-resistant bacteria (ARB), and antibiotic resistance genes (ARGs) associated with mobile genetic elements (MGEs) (1–5). Recently, several foodborne disease outbreaks have been associated with produce contamination worldwide (5–9). The microbiome of produce is important for plant health and vigor and was shown to be highly dynamic during growth and postharvest (10), but can also contain potentially pathogenic bacteria from human and animal sources, including Escherichia coli strains (11). Contamination can occur preharvest (i.e., through organic fertilizers, soil, wild animals, or contaminated irrigation water) and postharvest (12, 13).

Antibiotic resistance in bacterial pathogens has increased globally due to the widespread use and misuse of antibiotics (14–17). Antibiotic resistance levels in E. coli are useful indicators of overall resistance levels of bacteria on foods and in animals and humans (11). Antibiotic resistance and ARGs have been documented for enteric bacteria from various types of produce, which could facilitate the dissemination of resistant bacteria to a wider community of people (1, 2, 4, 16,18, 19). If ARGs are localized on MGEs such as plasmids or conjugative transposons they can be transferred horizontally to pathogens (20). Horizontal gene transfer (HGT) takes place at sites with high cell densities of plasmid donors and recipients, nutrient availability, and selective pressure. The phytosphere, including the rhizosphere and the phyllosphere, have been reported as hot spots of HGT (21). The plasmid-mediated resistome of produce bacteria might provide the enterobacteria with ARGs in the intestine under selective conditions. Conjugative plasmids can often confer resistance not only toward multiple antibiotics but also toward heavy metal compounds or disinfectants, making coselection possible (22–25). Although plasmids belonging to the incompatibility groups IncF and IncI have a narrow host range (NHR), they are assumed to be important for the dissemination of ARGs in E. coli and other Enterobacteriaceae (26, 27). Most importantly, resistance- and virulence-associated traits of E. coli isolates were almost exclusively found on IncF group plasmids (28–30). However, no real-time PCR (RT-PCR) systems that allow the cultivation-independent detection and quantification of these plasmids in total community DNA (TC-DNA) are available.

**FIG 1:**
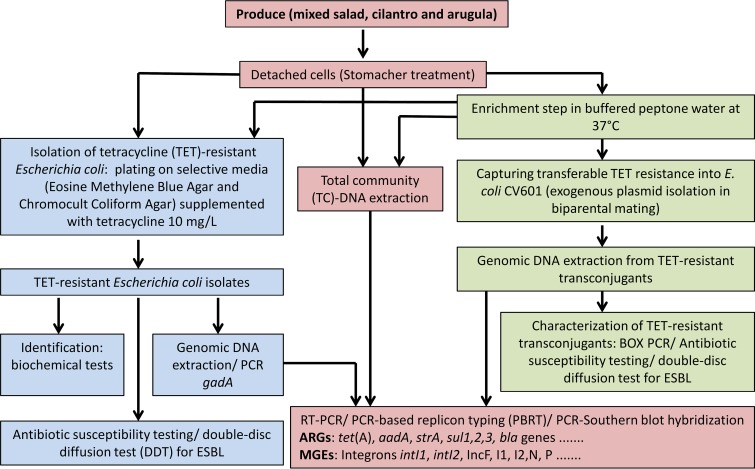
Flow diagram of the experimental setup of this study to evaluate the use of culture-dependent and -independent approaches to characterize the transferable resistome of bacteria associated with produce.

In this study, culture-dependent and -independent approaches were employed to assess the transferable resistome of bacteria associated with produce (Fig. 1). We focused on tetracycline (TET) resistance because of the large amounts of tetracyclines used in animal husbandries resulting in a high load released into the agro-ecosystem via organic fertilizers (31). TET-resistant E. coli was isolated from produce directly after purchase and after seven days of storage by selective plating with and without prior nonselective enrichment. In addition, transferable TET resistance plasmids were captured into E. coli recipient strains using the so-called exogenous plasmid isolation method (32). New real-time PCR primers were developed for the detection and quantification of IncF and IncI plasmids. TC-DNA was also extracted from the microbial fraction detached from produce or after nonselective enrichment to detect and quantify the abundance of ARGs and MGEs.

## RESULTS

### Phenotypic and genotypic characterization of TET-resistant E. coli isolates.

To find out whether produce was a source of antibiotic-resistant E. coli, we determined the occurrence and resistance profiles of TET-resistant E. coli isolated from 24 samples of produce directly or after an overnight enrichment step. The phenotypic characterization of a total of 63 TET-resistant E. coli isolates from cilantro (*n* = 54), arugula (*n* = 7), and mixed salad (*n* = 2), of which 50 were recovered after nonselective enrichment and 13 without enrichment (20.6%) revealed an impressive diversity (Table 1).

**TABLE 1:** Characterization of representative tetracycline-resistant *E. coli* isolates from produce

E. coli isolates^*a*^	Sample source^*b*^	Time point (day)	Inc group^*c*^	*bla* genes	Resistance and integrase genes	Antibiotic resistance profile^*d*^
EK2.1^D^	Ci	0	FII^l^	*bla*_TEM_	*tet*(A), *qnrS*	AM, AMX, TET, CIP, OFX
EK2.2^E^	Ci	0	U^h^	*bla*_TEM_	*intI1*, *tet*(A), *sul2*, *strA*, *qnrS*	AM, AMX, TET, S, TMP, SD, CIP, OFX
EK2.5^D^	Ci	0	I1^l^	*bla*_TEM_	*tet*(A), *qnrS*	AM, AMX, TET, CIP
EK2.7^D^	Ci	0	U^h^	*bla*_TEM_	*intI1*, *tet*(A), *sul2*, *strA*, *qnrS*	AM, AMX, TET, S, TMP, SD, OFX
EK2.8^D^	Ci	0	I1^l^	*bla*_TEM_	*intI1*, *tet*(A), *sul2*, *strA*	AM, AMX, TET, S, TMP
EK2.11^D^	Ci	0	I1^f^	*bla*_TEM_	*tet*(A)	AM, AMX, TET
EK2.15^D^	Ci	0	I1^f^	*bla*_TEM_	*tet*(A), *sul2*	AM, AMX, TET
EK2.16^D^	Ci	0	N^g^	*bla*_TEM_	*tet*(A), *sul2*, *sul3*, *strA*	AM, AMX, TET, S
EK2.18^D^	Ci	0	X1^h^	*bla*_TEM_	*tet*(A), *sul2*, *sul3*, *strA*	AM, AMX, TET, S
EK2.19^E^	Ci	0	FII^l^	*bla*_TEM_	*intI1*, *tet*(A), *merRTΔP*, *sul2*, *aadA*	AM, AMX, TET, S, TMP, D, GM, KM
EK2.20^E^	Ci	0	HI1^h^	*bla*_TEM_	*intI1*, *tet*(A), *sul2*, *sul3*, *aadA*, *qnrS*	AM, AMX, TET, S, TMP, SD, GM, OFX, C
EK2.21^E^	Ci	0	X1^h^	*bla*_TEM_	*intI1*, *tet*(A)	AM, AMX, TET, OFX
EK2.22^E^	Ci	0	X1^h^	*bla*_TEM_	*intI1*, *tet*(A), *strA*, *qnrS*	AM, AMX, TET, S, TMP, SD, OFX
EK2.25^E^	Ci	0	U^h^, X1^h^	*bla*_TEM_	*intI1*, *tet*(A), *sul2*, *strA*, *qnrS*	AM, AMX, TET, S, TMP, SD, CIP, OFX
EK2.26^E^	Ci	0	U^h^	*bla*_TEM_	*intI1*, *tet*(A), *sul1*, *aadA*, *qacE/qacEΔ1*, *strA*	AM, AMX, TET, S, TMP, SD, CIP, NA, OFX
EK2.29^E,k^	Ci	0	N^g^	*bla*_TEM_, *bla*_CTX-M-1_	*intI1*, *tet*(A), *sul1*, *strA*, *qnrS*	AM, AMX, TET, S, TMP, SD, CRO, CTX, OFX, CIP
EK2.30^E^	Ci	0	U^h^	*bla*_TEM_	*intI1*, *tet*(A), *sul1*, *sul3*, *aadA*, *qnrS*	AM, AMX, TET, TMP, SD, C
EK3.33^E^	Ci	0	X1^h^		*intI1*, *sul1*, *strA*	TET, D
EK3.34^D^	Ci	0	FII^l^	*bla*_TEM_	*intI1*, *tet*(A), *sul1*, *strA*	AM, AMX, TET, S, TMP, SD, NA, OFX
EK3.35^D^	Ci	0	FII^l^	*bla*_TEM_	*tet*(A), *sul1*	AM, AMX, TET, S, TMP, SD, NA
EK3.36^E^	Ci	0	FIB^l^	*bla*_TEM_	*intI1*, *tet*(A), *merRTΔP*, *sul1*, *aadA*	AM, AMX, TET, S, TMP, D, GM, KM
EK3.43^E,k^	Ci	0	N^g^	*bla*_CTX-M-1_	*tet*(A), *sul1*, *qnrS*	AM, AMX, TET, S, D, GM, CTX, OFX
EK3.44^E,k^	Ci	7	N^g^	*bla*_TEM_, *bla*_CTX-M-1_	*intI1*, *tet*(A), *merRTΔP*, *sul1*, *strA*, *qnrS*	AM, AMX, TET, S, TMP, SD, CRO, CTX, C, NA, CIP, OFX
EK5.16^E^	Ci	7	FII^l^, I1^f^	*bla*_TEM_	*intI1*, *tet*(A), *aadA*, *qacE/qacEΔ1*, *qnrS*	AM, AMX, TET, S, TMP, D, OFX
EK5.19^E^	Ci	7	X1^h^	*bla*_TEM_	*intI1*, *tet*(A), *sul2*, *strA*, *qnrS*	AM, AMX, TET, S, TMP, SD, D, C
EK5.20^E^	Ci	7	FII^l^, I1^f^	*bla*_TEM_	*intI1*, *tet*(A), *aadA*, *qacE/qacEΔ1*, *qnrS*	AM, AMX, TET, S, TMP, OFX
EK5.25^E^	Ci	7	FII^l^	*bla*_TEM_	*intI1*, *tet*(A), *qacE/qacEΔ1*, *qnrS*	AM, AMX, TET, TMP, OFX
EK5.28^E^	Ci	7	FII^l^, I1^f^	*bla*_TEM_	*intI1*, *tet*(A), *aadA*, *qacE/qacEΔ1*, *qnrS*	AM, AMX, TET, S, TMP, CIP, OFX
EK5.30^E^	Ci	7	FII^l^, FIB^l^, I1^f^	*bla*_TEM_	*intI1*, *tet*(A), *sul1*, *aadA*, *qacE/qacEΔ1*, *qnrS*	AM, AMX, TET, TMP, SD, D, CIP, NA, OFX
EK5.32^E^	Ci	7	X1^h^	*bla*_TEM_	*intI1*, *tet*(A), *sul1*, *sul2*, *strA*, *qnrS*	AM, AMX, TET, S, TMP, SD, OFX, C
EK5.40^E^	Ci	7	X1^h^	*bla*_TEM_	*intI1*, *tet*(A), *sul2*, *strA*, *qnrS*	AM, AMX, TET, S, TMP, SD, C
EK7.6^E^	Ci	7	U^h^	*bla*_TEM_	*intI1*, *tet*(A)	AM, AMX, TET, TMP, D
EK7.7^E^	Ci	7	I1^f^	*bla*_TEM_	*intI1*, *tet*(A), *qacE/qacEΔ1*, *aadA*	AM, AMX, TET, TMP
EK7.9^E^	Ci	7	X1^f^	*bla*_TEM_	*intI1*, *tet*(A), *merRTΔP*, *sul2*, *qnrS*	AM, AMX, TET, SD, CRO, D, CIP, C, GM, OFX
EK7.10^E^	Ci	7	HI1^h^	*bla*_TEM_	*intI1*, *tet*(A), *merRTΔP*, *sul2*, *qnrS*	AM, AMX, TET, SD, CRO, D, CIP, C, GM, OFX
EK7.11^E^	Ci	7	FII^l^, I1^f^	*bla*_TEM_	*intI1*, *tet*(A), *aadA*, *qacE/qacEΔ1*, *qnrS*	AM, AMX, TET, TMP, CIP
EK7.12^E^	Ci	7	FII^l^, I1^f^	*bla*_TEM_	*intI1*, *tet*(A), *aadA*, *qacE/qacEΔ1*, *qnrS*	AM, AMX, TET, TMP
EK7.13^E^	Ci	7	FIB^l^, I1^f^	*bla*_TEM_	*intI1*, *tet*(A), *aadA*, *qacE/qacEΔ1*, *qnrS*	AM, AMX, TET, TMP, OFX
EK7.14^E^	Ci	7	FII^l^	*bla*_TEM_	*intI1*, *aadA*, *qacE/qacEΔ1*, *qnrS*	AM, AMX, TET, TMP
EK7.15^E^	Ci	7	FII^l^	*bla*_TEM_	*intI1*, *tet*(A), *sul1*, *sul2*, *aadA*, *qacE/qacEΔ1*, *qnrS*	AM, AMX, TET, S, TMP, SD, CIP, GM
EK7.16^E^	Ci	7	FII^l^	*bla*_TEM_	*intI1*, *tet*(A), *sul1*, *sul2*, *strA*, *qacE/qacEΔ1*, *qnrS*	AM, AMX, TET, S, TMP, SD, CIP, OFX
EK7.17^E^	Ci	7	FII^l^, X1^h^	*bla*_TEM_	*tet*(A), *qnrS*	AM, AMX, TET, CIP, OFX
EK7.18^E^	Ci	7	FII^l^	*bla*_TEM_	*intI1*, *tet*(A), *sul2*, *strA*, *qnrS*	AM, AMX, TET, S, TMP, SD, CIP, OFX
EK7.19^E^	Ci	7	FIB^l^, I1^f^	*bla*_TEM_	*intI1*, *tet*(A), *aadA*, *qacE/qacEΔ1*, *qnrS*	AM, AMX, TET, TMP
EK7.21^E^	Ci	7	FIB^l^, I1^f^	*bla*_TEM_	*intI1*, *tet*(A), *aadA*, *qacE/qacEΔ1*, *qnrS*	AM, AMX, TET, TMP, OFX
EK7.22^E^	Ci	7	FII^l^, FIB^l^	*bla*_TEM_	*intI1*, *tet*(A), *sul1*, *sul2*, *sul3*, *aadA*, *strA*, *qacE/qacEΔ1*, *qnrS*	AM, AMX, TET, S, TMP, SD, CIP, GM, NA, OFX
EK7.24^E^	Ci	7	FII^l^, I1^f^	*bla*_TEM_	*intI1*, *tet*(A), *sul1*, *sul2*, *aadA*, *strA*, *qacE/qacEΔ1*, *qnrB*, *qnrS*	AM, AMX, TET, S, TMP, SD, CIP, OFX, C
EK7.26^E^	Ci	7	FIB^l^, I1^f^	*bla*_TEM_	*intI1*, *tet*(A), *aadA*, *qacE/qacEΔ1*, *qnrS*	AM, AMX, TET, TMP, CIP, OFX
EK7.27^E^	Ci	7	X1^h^	*bla*_TEM_	*tet*(A), *merRTΔP*, *sul2*, *qnrS*	AM, AMX, TET, SD, CRO, D, CIP, C, GM, OFX
EK7.28^E^	Ci	7	FIB^l^, I1^f^	*bla*_TEM_	*intI1*, *tet*(A), *aadA*, *qacE/qacEΔ1*, *qnrS*	AM, AMX, TET, TMP
EK7.29^E^	Ci	7	FII^l^, FIB^l^	*bla*_TEM_	*intI1*, *tet*(A), *sul1*, *aadA*, *qacE/qacEΔ1*, *qnrS*	AM, AMX, TET, S, TMP, SD, D, CIP, NA, OFX
EK7.30^E^	Ci	7	FII^l^	*bla*_TEM_	*intI1*, *tet*(A), *sul1*, *sul2*, *aadA*, *qacE*/*qacEΔ1*, *qnrS*	AM, AMX, TET, S, TMP, SD, GM
EK6.1^E^	Ci	7	FII^l^		*intI1*, *tet*(A), *sul2*, *strA*	TET, S
EK6.3^E^	Ci	7	FIB^l^		*tet*(A), *sul2*, *strA*	TET, S
M1^E^	MS	0	ND	*bla*_TEM_	*intI1*, *tet*(A), *sul1*, *sul2*, *qacE/qacEΔ1*, *strA*, *merRTΔP*	AM, AMX, TET, S, TMP, SD, D
M19^E^	MS	0	ND	*bla*_TEM_	*intI1*, *tet*(A), *sul1*, *sul2*, *qacE/qacEΔ1*, *strA*	AM, AMX, TET, S, TMP, SD, D
RE1^D^	A	0	FII^l^			TET, D
RE4^D^	A	0	I1^l^		*intI1*, *tet*(A), *sul3*, *aadA*, *merRTΔP*	TET, S, TMP, SD, D, CIP, NA
RE9^D^	A	0	I1^l^		*intI1*, *tet*(A), *sul3*, *aadA*, *merRTΔP*	TET, S, TMP, SD, D, CIP, NA
RE10^D^	A	0	I1^f^		*intI1*, *tet*(A), *sul3*, *aadA*, *merRTΔP*	TET, S, TMP, SD, D, CIP, NA
RE11^E^	A	0	I1^f^		*intI1*, *tet*(A), *sul3*, *aadA*, *merRTΔP*	TET, S, TMP, SD, D, CIP, NA
RE14^E^	A	7	FII^l^			TET, D
RE19^E^	A	7	I1^f^		*intI1*, *tet*(A), *sul3*, *aadA*, *merRTΔP*	TET, S, TMP, SD, D, CIP, NA

a D, direct plating; E, enrichment.

b Ci, cilantro; MS, mixed salad; A, arugula.

c l, detected by RT-PCR and PBRT; f, detected by RT-PCR; g, detected by PCR; h, detected by PBRT; k, conjugal transfer into *E. coli* CV601; ND, not detected.

d AM, ampcillin; AMX, amoxicillin; TET, tetracycline; CIP, ciprofloxacin; OFX, ofloxacin; S, streptomycin; TMP, trimethoprim; SD, sulfadiazine; D, doxycycline; GM, gentamicin; KM, kanamycin; C, chloramphenicol; NA, nalidixic acid; CRO, ceftriaxone; CTX, cefotaxime.

Almost all E. coli isolates were resistant to antibiotics from at least one class, and two isolates were resistant to eight antibiotic classes, tetracyclines (TET and D), penicillins (AM and AMX), third generation cephalosporins (CTX and CRO), fluoroquinolones (CIP, OFX, and NA), aminoglycosides (GM and S), sulfonamides (SD), phenicols (C), and trimethoprim (TMP). Most of the TET-resistant E. coli also displayed resistance to ampicillin and amoxicillin (84%) and trimethoprim (73%). Resistances to ofloxacin, ciprofloxacin, sulfadiazine, and streptomycin were also common. We tested all of the isolates for the production of extended-spectrum beta-lactamases (ESBLs) with the double-disc diffusion test (DDT) and found three ESBL-producing E. coli which were isolated from two of the cilantro samples.

We then genotypically characterized the collection of E. coli isolates for the presence of various resistance genes [*tet*(A), *strA*, *sul1*, *sul2*, *sul3*, *aadA*, *qacE* and/or *qacEΔ*1 (*qacE*/*qacEΔ*1), *merRTΔP*, *bla* genes (TEM, CTX-M, and SHV), *qnr* genes (*qnrA*, *qnrB*, and *qnrS*)] and integrase genes *intI1* and *intI2* by RT-PCR or regular PCR of genomic DNA (Table 1). The most commonly detected ARG was the tetracycline resistance gene *tet*(A), which was found in 59 out of 63 isolates. A total of 10 isolates were positive for the sulfonamide resistance genes *sul1*, 14 for *sul2*, and five for *sul3*. The combinations of *sul1* and *sul2*, *sul2* and *sul3*, and *sul1* and *sul3* were detected in seven, three, and one isolate, respectively. All three *sul* genes were found in one TET-resistant E. coli isolate from cilantro. The *qnrB* and *qnrS* genes encoding fluoroquinolone resistance were detected alone or in combination in one and 38 isolates, respectively. The *bla*_TEM_ genes encoding resistance to ampicillin and amoxicillin were detected in 82.5% of TET-resistant E. coli isolates. The *bla*_CTX-M-1_ gene encoding ESBL resistance was detected in only three isolates and was found in combination with *bla*_TEM_ genes in two E. coli from cilantro. The *bla*_SHV_ gene encoding ESBL resistance was not detected in any of the isolates. For the streptomycin/spectinomycin resistance genes, *aadA* (24 isolates) was most common, followed by *strA* (21 isolates) and *aadA* and *strA* (three isolates).

The class 1 integron integrase gene *intI1* was detected in 50 isolates, while the class 2 integron integrase gene *intI2* was not detected at all. Although *qacEΔ*1 encoding quaternary ammonium compound resistance is a typical component of class 1 integrons, the *qacE* and/or *qacEΔ*1 genes were detected in only 23 isolates, suggesting a large proportion of atypical class 1 integrons. Interestingly, *merRTΔP* encoding regulation, transport, and extracellular mercury-binding was detected in 12 isolates. These findings show that produce can be a source of multidrug-resistant E. coli isolates.

### Characterization of plasmids in TET-resistant E. coli isolates.

To test if the TET-resistant E. coli isolates recovered from produce harbor plasmids and to assign them to known plasmid groups, their genomic DNA was screened by TaqMan probe-based RT-PCR systems for IncF and IncI plasmids and by PCR-based replicon typing (PBRT) (Table 1). All isolates that were positive by RT-PCR targeting the IncF (*traI* gene) were also identified by replicon typing as IncF, confirming the specificity of the novel TaqMan RT-PCR system. However, PBRT also allowed assignment to the different IncF subgroups. Furthermore, other plasmids were also identified by PBRT or RT-PCR (*korB*, specific for IncP-1 plasmids) or PCR (IncN). A summary of the plasmid/replicon types detected among the 63 representative TET-resistant E. coli isolates is given in Table 1. For cilantro and arugula, almost all TET-resistant E. coli isolates contained plasmids (61 out of 63), but the plasmids detected in the two isolates from mixed salad could not be assigned using RT-PCR or PBRT. In most isolates (*n* = 45), one plasmid type was detected, but some had two (*n* = 15) or three (*n* = 1) plasmids. Plasmids from seven different Inc groups were found in the 63 E. coli isolates, IncFII (*n* = 21), IncI1 (*n* = 17), IncX1 (*n* = 11), IncFIB (*n* = 10), IncU (*n* = 6), IncN (*n* = 4), and IncHI1 (*n* = 2). All Inc groups were found in E. coli isolates from cilantro, whereas only two Inc groups were found in isolates from arugula, IncI1 (*n* = 5) and IncFII (*n* = 2). Plasmids of the IncF groups (FII and FIB) were the predominant types, followed by IncI1 and IncX1 plasmids. The combination of replicon types IncFII and IncFIB was detected in two isolates, whereas the combination of replicon types IncFII and IncI1 and the combination of IncFIB and IncI1 were found in six and five isolates, respectively. In one isolate from cilantro, the combination IncFII, IncFIB and IncI1 was detected. IncI2 plasmids were not detected in any of the E. coli isolates.

### Conjugal transfer of antibiotic resistance.

Conjugation experiments were conducted in order to determine the potential transfer of antibiotic resistances to other bacteria. Conjugal transfer experiments were performed using TET-resistant E. coli isolates positive for ESBL (EK2.29, EK3.43, and EK3.44) as donors and kanamycin- and rifampin-resistant E. coli CV601 as a recipient at 37°C. We selected transconjugants on LB plates containing tetracycline and cefotaxime, which corresponded to phenotypes of the donors. The transfer of the resistance phenotypes was successful.

### Phenotypic and genotypic characterization of plasmids captured via exogenous isolation.

We further investigated the presence of transferable plasmids in produce by capturing TET resistance plasmids from nonselective enrichment cultures of fresh leaves from cilantro, mixed salad, or arugula by exogenous plasmid isolation into E. coli CV601. TET-resistant transconjugants were captured only on day 0 but not on day 7. The transfer frequencies of TET-resistant transconjugants were 1.73 × 10^−7^, 1.55 × 10^−4^, and 4.66 × 10^−9^ per recipient in cilantro, mixed salad, and arugula, respectively. While all transconjugants obtained from cilantro (*n* = 27) and arugula (*n* = 23) were characterized, a total of only 41 transconjugants from mixed salad was analyzed due to the high number of transconjugants obtained. Based on initial phenotypic and genotypic analyses, 15 representative out of 91 TET-resistant transconjugants from produce (cilantro, *n* = 12; arugula, *n* = 1; mixed salad, *n* = 2) were selected for further characterization. The majority of these transconjugants acquired resistance to at least two antibiotic classes, and all were resistant to tetracycline, ampicillin, and amoxicillin. The *bla*_TEM_ genes encoding ampicillin and amoxicillin resistances were detected in 86.7% of TET-resistant transconjugants (Table 2). The tetracycline resistance gene *tet*(A) was found in 13 out of 15 transconjugants from cilantro and arugula but not from mixed salad, while *tet*(Q) was detected in only one plasmid (pBMS1) isolated from the mixed salad. Four tetracycline resistance plasmids (pBC1.1, pBC1.3, pBC1.9, and pBC1.12) captured from cilantro carried the insertion sequence IS*1071*, class 1 integrons (*intI1*) and tetracycline resistance gene *tet*(A), but also encoded resistance to ampicillin (*bla*_TEM_), and mercury compounds (*merRTΔP*). Eight plasmids from cilantro transconjugants (pBC2.1, pBC2.2, pBC2.3, pBC2.4, pBC2.6, pBC2.8, pBC2.11, and pBC2.15) carried *tet*(A), *qnrS*, and *bla*_TEM_ and two of the plasmids (pBC2.1 and pBC2.4) carried in addition *sul1* and *sul2*, respectively. Two TET resistance plasmids (pBMS1 and pBMS4) captured from mixed salad carried *sul1*, *strA*, *merRTΔP*, *bla*_TEM_, and *intI1*. One plasmid (pBA1) captured from arugula carried *bla*_TEM_ and *tet*(A) (Table 2). Thus, this approach demonstrates that transferable multidrug resistance plasmids were easily captured by E. coli CV601, a process that might also occur in the human gut.

**TABLE 2:** Characterization of representative tetracycline resistant *E. coli* CV601 transconjugants captured from produce

TET^r^ E. coli CV601 transconjugants^*a*^	Sample source^*b*^	Inc groups^*c*^	*bla* genes	Resistance, integrase genes and IS^*d*^	Antibiotic resistance profile^*e*^
pBC1.1	Ci	P-1β^f^	*bla*_TEM_	*intI1*, *tet*(A), *merRTΔP*, *qacE/qacEΔ1*, IS*1071*	TET, AM, AMX, D
pBC1.3	Ci	P-1β^f^		*intI1*, *tet*(A), *merRTΔP*, *qacE/qacEΔ1*, IS*1071*	TET, AM, AMX, D
pBC1.9	Ci	P-1β^f^, FII^l^	*bla*_TEM_	*intI1*, *tet*(A), *merRTΔP*, *qacE/qacEΔ1*, IS*1071*	TET, AM, AMX, D
pBC1.12	Ci	P-1β^f^, FII^l^		*intI1*, *tet*(A), *merRTΔP*, *strA*, *qacE/qacEΔ1*, IS*1071*	TET, AM, AMX, D, S
pBC2.1	Ci	FIB^l^	*bla*_TEM_	*tet*(A), *sul1*, *qnrS*	TET, AM, AMX, D, CIP, NA, OFX, C
pBC2.2	Ci	FIB^l^	*bla*_TEM_	*tet*(A), *qnrS*	TET, AM, AMX, D, CIP, OFX
pBC2.3	Ci	FIB^l^, I1^f^	*bla*_TEM_	*tet*(A), *qnrS*	TET, AM, AMX, D, CIP, OFX
pBC2.4	Ci	FIB^l^	*bla*_TEM_	*tet*(A), *sul2*, *qnrS*	TET, AM, AMX, D, CIP, NA, OFX
pBC2.6	Ci	FIB^l^	*bla*_TEM_	*tet*(A), *qnrS*	TET, AM, AMX, D, CIP, OFX, C
PBC2.8	Ci	FIB^l^	*bla*_TEM_	*tet*(A), *qnrS*	TET, AM, AMX, D, CIP, NA, OFX
pBC2.11	Ci	FII^l^	*bla*_TEM_	*tet*(A), *qnrS*	TET, AM, AMX, D, CIP, OFX
pBC2.15	Ci	I1^f^	*bla*_TEM_	*tet*(A), *qnrS*	TET, AM, AMX, D, CIP, OFX
pBMS1	MS	FII^l^	*bla*_TEM_	*intI1*, *tet*(Q), *sul1*, *strA*, *merRTΔP*	TET, AM, AMX, D, TMP, C, S, SD
pBMS4	MS	FII^l^	*bla*_TEM_	*intI1*, *sul1*, *strA*, *merRTΔP*,	TET, AM, AMX, D, TMP, C, S, SD
pBA1	A	ND	*bla*_TEM_	*tet*(A)	TET, AM, AMX, D, TMP, C, CIP
E. coli CV601 (recipient)					

a Superscript r indicates resistance to the antibiotic.

b Ci, cilantro; MS, mixed salad; A, arugula.

c l, detected by RT-PCR and PBRT; f, detected by RT-PCR; ND, not detected.

d IS, insertion sequence.

e TET, tetracycline; AM, ampicillin; AMX, amoxicillin; D, doxycycline; S, streptomycin; CIP, ciprofloxacin; NA, nalidixic acid; OFX, ofloxacin; C, chloramphenicol; SD, sulfadiazine.

### Identification of exogenously isolated plasmids.

The newly developed TaqMan probe-based RT-PCR assay was used to screen the TET-resistant transconjugants for the presence of IncF and IncI plasmids and was validated by PBRT. In addition, other plasmids were also identified by RT-PCR (*korB*, specific for IncP-1 plasmids) and Southern blot hybridization. Plasmids of known Inc groups were detected in all transconjugants from the mixed salad and cilantro but not in the transconjugants from arugula. Representative transconjugants from cilantro and mixed salad carried either one (*n* = 11) or two (*n* = 3) replicons. In 12 transconjugants from cilantro samples, four different plasmid replicon types were detected (Table 2), IncFII (*n* = 3), IncFIB (*n* = 6), IncI1 (*n* = 2), and IncP-1β (*n* = 4). In contrast, the transconjugants isolated from mixed salad showed only one replicon type, IncFII (*n* = 2). One plasmid that could not be assigned by PBRT or RT-PCR was isolated from arugula leaves. The combination of replicon types IncFII and IncP-1β was detected in two transconjugants (pBC1.9 and pBC1.12), while the combination of replicon types of plasmids IncFIB and IncI1 was found in one transconjugant (pBC2.3) captured from cilantro leaves. Southern blot hybridization for sequences specific for IncP-1 plasmids revealed that four plasmids belonged to the IncP-1β subgroup. IncI2 plasmids were not detected in any TET-resistant transconjugants (Table 2). In contrast to IncFIB/FII and IncI1 plasmids, the IncP-1β plasmids captured exogenously were not detected in the 63 TET-resistant E. coli isolates.

### Detection of IncF and IncI plasmids, *tet*(A), and *intI1* in total community DNA.

We also screened for plasmids (IncF, IncI1, and IncI2), tetracycline resistance gene *tet*(A), and integrase gene *intI1* in TC-DNA extracted from bacterial communities either directly after their detachment from fresh leaves or after an enrichment step, using PCR-Southern blot hybridization and RT-PCR (Table 3). Using the RT-PCR method, IncF and IncI plasmids as well as the *tet*(A) gene were detected in TC-DNA extracted from enrichment cultures of leaves, but not in TC-DNA from the detached bacteria. In contrast, the *intI1* gene was detected in both kinds of TC-DNA. Consistent with these results, PCR-Southern blot hybridization targeting the IncF and IncI plasmids and *tet*(A) revealed strong hybridization signals in TC-DNA extracted from the enrichment cultures but very weak or no signals from direct extractions.

**TABLE 3:** PCR hybridization and real-time PCR of IncF, IncI1, and IncI2 plasmids and *intI1* and *tet*(A) from TC-DNA extracted from produce before and after enrichment^*a*^

Produce	DNA isolation	Time point (day)	IncF		IncI1		IncI2		*intI1*		*tet*(A)	
			RT-PCR	Blot	RT-PCR	Blot	RT-PCR	Blot	RT-PCR	Blot	RT-PCR	Blot
Mixed salad	Direct extraction	0	−	−	−	−	−	+++	+	(+++)	−	(++)^1^
		7	−	−	−	−	−	+++	+	(+++)	−	(+++)^2^
	Enrichment	0	+	(+++)	+	(++)	+	+++	+	(+++)	+	(++)^2^
		7	+	(+++)	+	(++)	+	+++	+	(+++)	+	+++
Arugula	Direct extraction	0	−	−	−	−	−	(++)^3^	+	(++)	−	(+++)^2^
		7	−	−	−	−	−	(++)^1^	+	(++)	−	(+++)^1^
	Enrichment	0	+	(+++)	−	−	+	+++	+	(++)	+	+++
		7	+	(+++)	−	−	+	+++	+	(++)	+	+++
Cilantro	Direct extraction	0	−	−	−	−	−	−	(+)^1^	(++)	(+)^1^	(+++)^3^
		7	(+)^1^	(++)^2^	−	−	−	−	(+)^3^	(+++)	(+)^2^	(+++)^3^
	Enrichment	0	(+)^2^	(+++)^2^	(+)^1^	(++)^2^	−	−	(+)^3^	(+++)	(+)^2^	(+++)^3^
		7	(+)^2^	(+++)^2^	(+)^2^	(++)^2^	−	−	(+)^3^	(+++)^3^	(+)^3^	(+++)^3^

a Superscript numbers indicate number of positive replicates; −, not detected or no signal; (+), positive (RT-PCR); (++), medium signal; (+++), strong signal.

## DISCUSSION

The present study showed that bacteria associated with produce can carry various plasmids that might represent an important link between the environmental and the human gut microbiomes. Although initially low in abundance, TET-resistant E. coli were isolated from all purchased produce samples after nonselective enrichment. Contamination of produce with E. coli strains can occur in the field through contaminated soil (organic fertilizers), exposure to contaminated irrigation water, or during postharvest (12, 13). In this study, TET-resistant E. coli isolates were mostly isolated from cilantro that was purchased from supermarkets in Braunschweig and Magdeburg, Germany, followed by mixed ready-to-eat salad and arugula purchased from supermarkets in Braunschweig. This suggests that produce might be a hot spot for contamination with E. coli carrying multidrug resistance plasmids that occur at low abundance. A high proportion of the TET-resistant E. coli isolates was also resistant to penicillins (AM and AMX) and trimethoprim. Although it is difficult to compare among studies because of different methodologies used for isolation and resistance testing, our results are in line with high resistance levels to penicillins and trimethoprim previously reported for E. coli from irrigation water and vegetables (18), ready-to-eat salads (1), and lettuce (2). In the present study, TET resistance was commonly conferred by *tet*(A), partly confirming previous studies reporting *tet*(A) and *tet*(B) genes as the most common TET resistance genes in E. coli and Salmonella spp. isolated from ready-to-eat vegetables (1, 33). The rapid dissemination of tetracycline resistance among bacteria has been related not only to the occurrence of TET resistance genes on transposons and conjugative plasmids (22, 23, 34), but also to selective pressure, e.g., the use of antibiotics in animal husbandry and the spread of TET resistance genes via organic fertilizers (31).

Plasmid-mediated multidrug resistance plays an important role in the transfer of ARGs around the world (35). Our study showed that E. coli isolates from produce harbored various plasmids belonging to replicon types IncF, IncI1, IncX1, IncU, IncN, and IncHI1, with IncF plasmids being the most frequently detected. These plasmids might play an important role in the dissemination of antibiotic resistances. IncF plasmids were found predominantly in E. coli isolated from drinking water (36) and poultry farms (37). In our study, IncFII was the most frequently detected replicon type (36.5%), followed by IncFIB (15.9%), which is in line with studies on E. coli recovered from pigs and humans (38), wastewater (39), and animals (40). The combination of replicon types IncFII and IncFIB in two isolates is consistent with a report on Enterobacter cloacae from lettuce (3). However, we cannot exclude that these replicons are located on the same plasmid, as several studies have reported the combination of replicon types as a multireplicon on a single plasmid (30, 41–43), likely due to cointegration (28). In this study, TET-resistant E. coli isolates which carried IncF plasmids were also positive for *tet*(A), *aadA*, *sul1*, *sul2*, *sul3*, *qacE* and/or *qacEΔ1*, *qnrB*, *qnrS*, or *bla*_TEM_ genes. Previous reports found that IncF plasmids can carry genes conferring resistance to all major antibiotic classes, including aminoglycosides, β-lactams, phenicols, tetracyclines, sulfonamides, and fluoroquinolones (38, 40, 44).

The NHR IncI1 plasmid types were the second most dominant replicon type (34.9%) and IncI1-positive isolates also carried multiple ARGs. In this study, strains carrying IncI1 plasmids were also positive for class 1 integron integrase gene *intI1* and a diverse set of resistance genes, namely *tet*(A), *sul2*, *strA*, *bla*_TEM_, *qacE* and/or *qacEΔ1*, *aadA*, *sul3*, *qnrS*, and/or *merRTΔP*. In a recent study, IncI1 plasmids from irrigation water and lettuce carried genes *sul1*, *tet*(A), *aadA*, *strA*, and *bla*_TEM_ as well as *intI1* (18). Similar phenotype and genotype profiles among E. coli strains from the current study and those recovered in previous studies from clinical samples, the environment, or other foods indicate that produce may play a potential role in the dispersal of E. coli carrying plasmid-localized ARGs. Thus, plasmids belonging to the IncF and IncI groups have the potential to be major contributors worldwide to the propagation of ARGs within enteric bacteria. One dissemination route of enteric bacteria carrying IncF and IncI plasmids might be the consumption of produce.

The newly developed TaqMan probe-based RT-PCR assays demonstrated high specificity in detecting these plasmids in E. coli isolates, and RT-PCR-positive isolates were also assigned by PBRT, which in addition enables subtyping.

This is the first study identifying NHR plasmids such as IncX1 and IncHI1 and broad-host-range (BHR) plasmid IncU in E. coli isolates recovered from cilantro leaves. Interestingly, IncX plasmids were detected in E. cloacae from lettuce (3). IncHI1 plasmids were previously reported in E. coli and Citrobacter youngae isolates from water and healthy calves, respectively (45), while the first IncU plasmids were isolated from Aeromonas salmonicida (46), and later from Aeromonas caviae from hospital effluent in the United Kingdom (47). In general, a low prevalence of ESBL-producing E. coli was found on produce, which is similar to previous studies (19, 48, 49). In the present study, ESBL-producing E. coli were isolated only from cilantro (2.8%).

To our knowledge, this is also the first report of E. coli isolates from cilantro that were positive for conjugative IncN plasmids, *bla*_CTX-M-1_, and resistance to third generation cephalosporins. The *bla*_CTX-M-1_ gene was also reported on plasmids belonging to the IncN family in E. coli isolated from farm workers, animals, humans, and the environment (50–52). Although IncN plasmids are able to replicate in a variety of Enterobacteriaceae, they are most frequently found in E. coli and Klebsiella pneumoniae, where they contribute to the dissemination of cephalosporin and carbapenem resistance (53).

The results of the present study showed that E. coli isolates harboring the *bla*_CTX-M-1_ gene also conferred resistance to at least seven classes of antibiotics tested. Moreover, E. coli harboring CTX-M genes were recently reported from lettuce and irrigation water (4, 54), raw vegetables (33, 54), and coastal waters (55, 56). Kim et al. (57) reported that ESBL-producing E. coli and Klebsiella pneumoniae carrying CTX-M were detected in ready-to-eat vegetables form a local retail market in Seoul, South Korea. A recent study has detected *bla*_TEM_ genes in association with IncF and IncI1 plasmids from irrigation water and lettuce from 16 household farms in Estarreja, Portugal (18).

In previous reports, the occurrence of *sul1* and *qacEΔ1* was frequently associated with class 1 integrons (3, 58). Unexpectedly, only 27% and 36.5% of *sul1*- and *qacE*/*qacEΔ1*-positive isolates carried the *intI1* gene, respectively, indicating that atypical class 1 integrons were more prevalent among the isolates, as previously also reported by Amos et al. (59).

In the present study, transferable TET resistance plasmids were also directly captured from the produce microbiomes on day 0 but not on day 7 after purchase, and the highest transfer frequency was observed in mixed salad, followed by cilantro and arugula. Differences in observed frequencies of transconjugants could be due to different abundances of bacteria with conjugative plasmids in the various sample types, or due to real differences in the frequencies of plasmid transfer. The latter might be affected by the metabolic activity of the produce microbiome, as plasmid transfer frequency is known to depend not only on plasmid-specific characteristics, but also on ecological factors affecting the metabolic activity of bacteria (60). Replicon types IncFII, IncFIB, IncI1, and IncP-1β were captured from cilantro leaves, whereas only IncFII plasmids were captured from mixed salad. IncF (FII and FIB) plasmids were prevalent among TET-resistant transconjugants from both types of produce. Most of the IncF plasmids exogenously captured harbored *bla*_TEM_, *tet*(A), and *qnrS* genes. One IncI1 plasmid was captured from cilantro, and another one was captured in combination with replicon type IncFIB. The conjugative plasmids carried *tet*(A) and *bla*_TEM_ genes. Finally, four IncP-1β plasmids were captured from cilantro leaves and two of them in combination with replicon type IncFII. IncP-1 plasmids have been frequently captured by exogenous plasmid isolation from various environments such as sewage sludge (61), manure (23), and water (62). However, the first isolations of IncP-1 plasmids were from clinical isolates (63, 64). The IncP-1β plasmids carried genes conferring resistances to antibiotics *tet*(A), *strA*, and *bla*_TEM_ and also mercury compounds (*merRTΔP*) and disinfectants (*qacE*/*qacEΔ1*).

In conclusion, this study showed that produce that we eat might contain bacteria such as E. coli carrying transferable multidrug resistance plasmids. Although E. coli numbers are typically low, our nonselective enrichments showed that proliferation can easily occur. Our study reports a specific TaqMan probe-based RT-PCR assay that can be used for rapid detection of IncF and IncI plasmids in E. coli isolates and exogenously captured plasmids as well as in TC-DNA extracted from enrichment cultures of leaves. However, quantifying these plasmids in TC-DNA directly extracted from the microbial fraction detached from leaves was impossible due to their low abundance in the microbiome, but IncF and IncI plasmids were detected in DNA extracted after previous enrichment. While these assays represent an important and useful tool to be implemented for monitoring the prevalence of IncF and IncI plasmids in isolates and the environment, negative results of these and other cultivation-independent methods can lead to an underestimation of the mobile resistome present in the rare microbiome of produce and other samples. This is the first study demonstrating that multidrug resistance plasmids present in produce-associated bacteria were transferable to sensitive E. coli recipients, a process that could occur in the human gut. The NHR plasmids IncF and IncI1 and also the BHR IncP-1β plasmids were captured from the produce. In particular, the captured IncF and Inc1 plasmids conferred resistance toward several classes of antibiotics. Thus, produce-associated bacteria should be considered an important route of disseminating transferable antibiotic resistances, which might be particularly relevant for patients under antibiotic treatment.

## MATERIALS AND METHODS

### Sample collection.

A total of 24 samples from different locally produced or imported produce (mixed salad, arugula, and cilantro) was analyzed. The mixed salad and arugula were purchased from local supermarkets in Braunschweig, Germany, in June and September 2016, and cilantro was obtained from supermarkets in Braunschweig and Magdeburg, Germany, in May 2017. The produce was stored at refrigerator temperature and sampled on days 0 and 7 (four replicates for each time point and produce type).

### Isolation and identification of TET-resistant E. coli.

For sampling, the produce was cut into pieces using a sterile scalpel and mixed. For each sample, 25 g each were filled in two stomacher bags (one for direct plating and the other for enrichment) and mixed three times with 75 ml buffered peptone water (BPW; Roth, Karlsruhe, Germany), with subsequent stomacher treatment performed with the Stomacher 400 (Seward, Worthing, United Kingdom) at high speed for 1 min. The enrichment cultures of fresh leaves in BPW were incubated at 37°C with shaking (150 rpm) for 18 to 24 h. In order to isolate TET-resistant E. coli, dilutions (10^−1^ and 10^−2^) of the sample suspensions and 100 µl of the enrichment cultures were plated on different culture media (eosin methylene blue [EMB]; Sifin, Berlin, Germany, and Chromocult coliform agar [CCA]; Merck, Darmstadt, Germany) supplemented with tetracycline (10 mg liter^−1^). All plates were incubated at 37°C for 18 to 24 h. The presumptive E. coli colonies were picked from each sample and streaked onto EMB, CCA, and TBX chromogenic agar (Roth, Karlsruhe, Germany) for confirmation by colony morphology and further characterization. E. coli isolates were then confirmed by biochemical tests for indole production, methyl red, and catalase activity (65). Furthermore, isolates were analyzed using PCR for the presence of the *gadA* gene encoding glutamate decarboxylase, specific for E. coli (66). E. coli isolates were stored in Luria broth (LB; Roth, Karlsruhe, Germany) containing 15% glycerol at −80°C.

### Exogenous plasmid isolation.

In order to capture tetracycline resistance plasmids, exogenous plasmid isolation via biparental mating was performed using gfp^+^-, kanamycin (Km)-, and rifampin (Rif)-resistant Escherichia coli CV601 (67) as a recipient. The recipient strain was grown overnight in tryptic soy broth (TSB; Merck, Darmstadt, Germany) supplemented with rifampin (Rif) (50 mg liter^−1^) and kanamycin (Km) (50 mg liter^−1^). Two milliliters of the recipient strain culture was transferred into a sterile Eppendorf tube and centrifuged at 3,100 × *g* for 5 min and washed twice with 1:10 TSB. Then, the pellet was resuspended in 2 ml of 1:10 TSB. The bacterial suspensions (donor) of each sample on days 0 and 7 were prepared from enrichment cultures of fresh leaves as described above. Twenty milliliters of each enrichment culture (donor) and 0.5 ml of recipient strain were mixed in a 50-ml falcon tube. As a background control, 5 ml of the enrichment cultures and 200 µl of the recipient were processed the same way as the samples. All mixtures were centrifuged at 3,100 × *g* for 10 min. The pellets were resuspended in 200 µl of 1:10 TSB and then spotted onto a filter for mating (Millipore filters, 0.22 µm). Filters were incubated overnight at 28°C on plate count agar plates (PCA; Merck, Darmstadt, Germany) supplemented with cycloheximide (Cyc) (100 mg liter^−1^). After incubation, the filters were placed in 2 ml of sterile 0.85% NaCl solution in a 50-ml falcon tube. Each filter was washed by vortexing for 1 min. Serial 10-fold dilutions were done and appropriate dilutions were plated on PCA agar supplemented with rifampin (Rif; 50 mg liter^−1^), kanamycin (Km; 50 mg liter^−1^), cycloheximide (Cyc; 100 mg liter^−1^), and tetracycline (TET; 15 mg liter^−1^) to select for tetracycline-resistant transconjugants. Background controls of bulk soil and the recipient controls were plated on the same selective media. Numbers of recipient cells were determined by applying three replicate 20-µl drops per each serial dilution (10^−5^ to 10^−8^) of all mating mixes on PCA with Km (50 mg liter^−1^), Rif (50 mg liter^−1^), and Cyc (100 mg liter^−1^). All plates were incubated at 28°C for up to 3 days. Transconjugants were determined by green fluorescence resulting from the green fluorescence protein (GFP). The identity of putative transconjugants was confirmed by BOX-PCR (68). Transfer frequencies were calculated as total number of transconjugants divided by the total number of recipients.

### Antibiotic susceptibility testing.

Antimicrobial susceptibility testing was performed by the disk diffusion method on Müller-Hinton agar (MH; Sigma-Aldrich, St. Louis, USA), according to the European Committee on Antimicrobial Susceptibility Testing (EUCAST). The antibiotics (µg) (Becton, Dickinson and Company, USA) used in this study were amoxicillin (25), ampicillin (10), cefotaxime (30), ceftazidime (30), ceftriaxone (30), chloramphenicol (30), ciprofloxacin (5), colistin (10), TET (30), doxycycline (30), streptomycin (10), gentamicin (10), ofloxacin (5), kanamycin (30), nalidixic acid (30), trimethoprim (5), and sulfadiazine (250). TET-resistant E. coli isolates were streaked onto LB agar supplemented with TET (10 mg liter^−1^), while TET-resistant E. coli CV601 transconjugants were streaked on plate count agar plates (PCA; Merck, Darmstadt, Germany) supplemented with TET (15 mg liter^−1^), Km (50 mg liter^−1^), and Rif (50 mg liter^−1^). E. coli strain CV601 was used as a negative control. The bacterial suspension was prepared from a single colony in normal saline (0.85% NaCl) to a density of 0.5 McFarland turbidity standard. Cotton swabs were used for streaking the suspension onto MH agar plates. After air drying, antibiotic discs were placed on the plates. Then all plates were incubated at 37°C for 18 to 24 h. The inhibition zone was measured. The results were interpreted according to the guidelines of EUCAST. Clinical and Laboratory Standards Institute (CLSI) recommendations were used when antibiotic breakpoints in EUCAST guidelines were absent (i.e., for doxycycline, streptomycin, tetracycline, and nalidixic acid). ESBL production was confirmed among TET-resistant E. coli isolates and transconjugants by double-disc diffusion test (DDT) (48). The ESBL producers were identified by phenotypic confirmatory test according to the CLSI.

### TC-DNA extraction.

The bacterial fraction detached from fresh leaves directly or after an enrichment culture of each sample as described above were pelleted by centrifugation at 3,100 × *g* for 15 min at 4°C. Total community DNA was extracted from the pellet using the FastDNA spin kit for soil (MP Biomedicals, Heidelberg, Germany), according to the manufacturer's instructions. The quality of extracted DNA was determined by agarose gel electrophoresis. The extracted DNA was stored at −20°C until further analysis.

### Genomic DNA extraction.

Genomic DNA was extracted from overnight cultures of TET-resistant E. coli isolates, transconjugants, and the recipient strain with a Qiagen genomic DNA extraction kit (Qiagen, Hilden, Germany) using a silica-based kit (silica bead DNA extraction kit; Thermo Scientific, St. Leon-Rot, Germany). The extracted genomic DNA was stored at −20°C until further analysis.

### Primer-probe design (IncF, IncI1, and IncI2 plasmids).

As it is known that relaxase genes can be used for classification of the mobilization systems of plasmids (69), the *traI* gene region was chosen as a target region to design primers detecting IncF, IncI1, and IncI2 plasmid sequences. A total of 4,530 plasmid DNA sequences were downloaded from NCBI (NCBI, Batch Entrez) using the 4,602 plasmid accession numbers found in GenBank by Shintani et al. (70), among which 298 plasmids were identified as belonging to the MOB_F_ group. The coding sequences (CDS) of the MOB_F_ plasmids were aligned using tBLASTn against the relaxase TraI of the F plasmid (GenBank accession number AP001918), resulting in 110 protein sequences sharing >50% identity and >70% coverage. The 110 protein sequences closely related to TraI were aligned using MAFT multiple sequence alignment software version 1.3.3. The alignment produced was back translated using the EMBOSS Backtranseq tool and used to generate a set of degenerated primers and probes using Primer3. All of those steps were carried out in Geneious 8.1.9. At best, 83 of the 110 *traI* nucleic acid sequences could be targeted by one set of designed primers and probe (Table 4). Those sequences belonged mostly to plasmids isolated from Salmonella enterica and Escherichia coli and a few from Klebsiella pneumoniae and Shigella spp. The plasmids corresponded to a part of the subclade MOB_F12_ defined by Garcillán-Barcia et al. (71), which comprises the phylogenetically broad IncF complex. When tested against the 4,530 plasmids, the primer/probe targeted 92 plasmids in the database, and 89 of these plasmids belonged to the MOB_F_ group (298 plasmids recovered belonged to this group) and three were annotated as “non-mob” and belonged to any MOB group. When looking at the “Inc” classification, 73 of the targeted plasmids belonged to the IncF (including FI and FII), 14 to the IncZ, and five to the pCD1 type. Among the 4,530 plasmid sequences, 243 carried a *rep* gene belonging to the IncF group, indicating that the primer/probe cannot detect all possible IncF plasmids.

**TABLE 4:** PCR and RT-PCR primer systems used in this study

Gene target	Primer	Primer and probe (5ʹ–3ʹ)	Size (bp)	Reference or source
*qacE* and/or *qacEΔ1*	qacEall-F qacEall-R	CGCATTTATTTTCTTTCTCTGGTT CCCGACCAGACTGCATAAGC	69	76
	qacEall-P	TGAAATCCATCCCTGTCGGTGT		
*tet*(A)	tetA-qfw tetA_qrv	CCGCGCTTTGGGTCATT TGGTCGCGTCCCAGTGA	504	77
	q-tetA-P	TCGGCGAGGATCG		
*sul1*	q-sul_1 653f q-sul_1 719r	CCGTTGGCCTTCCTGTAAAG TTGCCGATCGCGTGAAGT	965	78
	tp_sul1	CAGCGAGCCTTGCGGCGG		
*sul2*	q_sul2 595f	CGGCTGCGCTTCGATT	865	78
	q_sul2 654f	CGCGCGCAGAAAGGATT		
	tp_sul2 614	CGGTGCTTCTGTCTGTTTCGCGC		
*sul3*	Sul3-F	CAGATAAGGCAATTGAGCATGCTCTGC	569	38
	Sul3-R	AGAATGATTTCCGTGACACTGCAATCATT		
*intI1*	intI1-LC1 intI1-LC5	GCCTTGATGTTACCCGAGAG GATCGGTCGAATGCGTGT	196	79
	intI1-P	ATTCCTGGCCGTGGTTCTGGGTTTT		
*aadA*	q-aadA-Fw q-aadA-Rv	TTGATTTGCTGGTTACTGTG CTTAGTGTGATCTCGCCTTT	635	80
	q-aadA-P	TGGTAGGTCCAGCGGCGGAG		
*korB*	korB-F	TCATCGACAACGACTACAACG	118	81
	korB-Fz	TCGTGGATAACGACTACAAACG		
	korB-R	TTCTTCTTGCCCTTCGCCAG		
	korB-Rge	TTYTTCYTGCCCTTGGCCAG		
	korB-Rd	TTCTTGACTCCCTTCGCCAG		
*strA*	q-strA-Fw q-strA-Rv	TCAATCCCGACTTCTTACCG CACCATGGCAAACAACCATA	521	80
	q-strA-P	TGCTCGACCAAGAGCGGC		
*intI2*	intI2-LC2 intI2-LC3	TGCTTTTCCCACCCTTACC GACGGCTACCCTCTGTTATCTC	195	79
	intI2-P	TGGATACTCGCAACCAAGTTATTTTTACGCTG		
*tet*(Q)	q-tetQ-Fw q-tetQ-Rv	AGGTGCTGAACCTTGTTTGATTC GGCCGGACGGAGGATTT	69	82
	q-tetQ-P	TCGCATCAGCATCCCGCTC		
IncF (*traI*)	682_F	CACGGTATGTGGGARATGCC	391	This study
	1073_R	TCCGGCGGCAGYATVCCRAC		
	973_P	CAGCAGGCGGTGRCRCAGGC		
IncI1 (*traI*)	IncI1_traI_Fwd	TTCTTCTTCCCCTACCATC	118	This study
	IncI1_traI_Rev	CATTTTCCAGCGTGTTTC		
	IncI1_traI_TP	CGGCTTTTCACTTCGTGGTT		
IncI2 (*traI*)	IncI2_traI_Fwd	CAAGAACAGAAACAGGCA	291	This study
	IncI2_traI_Rev	TCCCGCAGATAACAGATA		
	IncI2_traI_TP	CCAAACCAACCACAACCA		
*merRTΔP*	merRT-P merRT-P	GGGAGATCTAAAGCACGCTAAGGCRTA GGGGAATTCTTGACWGTGATCGGGCA	1000	83
*bla*_CTX-M-1_	CTX-M-F	TCTTCCAGAATAAGGAATCCC	908	84
	CTX-M-R	CCGTTTCCGCTATTACAAAC		
*bla*_TEM_	TEM-F	TCCGCTCATGAGACAATAACC	930	84
	TEM-R	TTGGTCTGACAGTTACCAATGC		
*bla*_SHV_	SHV-F	TTATCTCCCTGTTAGCCACC	796	84
	SHV-R	GATTTGCTGATTTCGCTCGG		
IS*1071*	IS-F	GCTTGGTCACTTCTGGGTCTTC	180	85
	IS-R	CTATGCCCGTCTATCGTTACCC		
IncN	rep-1	AGTTCACCACCTACTCGCTCCG	165	86
	rep-2	CAAGTTCTTCTGTTGGGATTCCG		
*qnrA*	qnrAf-RT	ATTTCTCACGCCAGGATTTG	529	87
	qnrAr-RT	GCAGATCGGCATAGCTGAAG		
*qnrB*	qnrBmF	GGMATHGAAATTCGCCACTG	429	87
	qnrBmR	TTYGCBGYYCGCCAGTCGAA		
*qnrS*	qnrSrtF11	GACGTGCTAACTTGCGTGAT	393	87
	qnrSrtR11	TGGCATTGTTGGAAACTTG		

Available IncI plasmid sequences were downloaded from NCBI. The *traI* genes were aligned using the software CLC Main Workbench version 8 (CLC bio, Qiagen) with standard settings for alignments, and primers were designed to match conserved regions of the *traI* gene (Table 4). The specificity was confirmed *in silico* with NCBI primer BLAST and with a set of plasmids from other incompatibility groups. Plasmids used for this test were R388, pB10, pHHV216, RSF1010, pSM1890, RP4, pHH3-414, pHH2-227, pRA3, RN3, RSF1010, pTH10, pTP6, R751, pQKH54, pKS208, pEST4002, pJKJ5, pMCBF1, pRMS149, pCAR1, pD2RT, pD67, pWW0, and pST527, from which none was amplified.

### Detection of IncF, IncI plasmids by real-time PCR.

The RT-PCR assay was performed under standard conditions. All real-time PCR (RT-PCR) reactions were set up in a 25 µl reaction volume using a Hot Start *Taq* DNA polymerase (M0495L, New England BioLabs, Ipswich, Massachusetts, USA) containing 5 µl of template DNA, 300 nM primer (reverse), 50 nM primer (forward), and 50 nM probe for IncF, and 300 nM primer each (forward and reverse) and TaqMan probe for IncI. All primers and probes are described in Table 4. The following PCR program was used for amplification: 10 min at 95°C, followed by 40 cycles of 95°C for 30 sec and 60°C for 1 min. The assays were carried out in triplicate with real-time PCR 5ʹ-nuclease assays (TaqMan RT-PCR) in a CFX96 real-time PCR detection system (Bio-Rad, Hercules, CA, USA). Negative controls were included in all tests, and they consisted of all the elements of the reaction except for the template DNA.

Standard plasmids were used to construct a full standard curve in duplicate in each RT-PCR run. Standard plasmids were constructed by cloning the purified PCR products amplified from the plasmids R64 for IncI1, pHNSHP45 for IncI2, and F plasmid for IncF using the corresponding primer pairs used for the RT-PCR, into TransforMax EC100 electrocompetent E. coli (Epicentre), in pJET1.2 using the Thermo Scientific CloneJET PCR cloning kit (Thermo Scientific).

### Detection of target genes by real-time PCR and PCR.

The target genes in genomic DNA extracted from TET-resistant E. coli isolates and transconjugants were detected by RT-PCR 5ʹ-nuclease assays (TaqMan or EvaGreen RT-PCR) in a CFX96 RT-PCR detection system (Bio-Rad, Hercules, CA, USA) or by PCR for class 1 and 2 integrons; integrase genes *intI1* and *intI2*; *korB* (IncP-1 plasmids); *qacE* and/or *qacEΔ1* (*qacE*/*qacEΔ1*) encoding quaternary ammonium compound resistance; *aadA* and *strA* encoding streptomycin and spectinomycin resistance; tetracycline resistance genes [*tet*(Q) and, *tet*(A)]; the *merRTΔP* gene part of the mercury resistance operon; *sul1*, *sul2*, and *sul3* encoding sulfonamide resistance; *qnrA*, *qnrB*, and *qnrS* encoding fluoroguinolone resistance; β-lactam resistance genes (*bla*_TEM_, *bla*_SHV_ and *bla*_CTX-M-1_); IncN (*rep*); and insertion sequence (IS) IS*1071* (represented by the *tnpA* gene). The DNA of the recipient strain E. coli CV601 was included as the negative control. The primers and probes targeting these genes and PCR and RT-PCR conditions are listed in Table 4.

### Conjugation assay.

TET-resistant E. coli isolates positive for ESBL (EK2.29, EK3.43, and EK3.44) were examined for their ability to transfer resistance. Briefly, the donors and the rifampin- and kanamycin-resistant E. coli CV601 recipient strain were grown in LB broth overnight at 37°C. 500 µl of overnight cultures of each donor and recipient strain were mixed in 1 ml LB broth and incubated at 37°C for 24 h without shaking. 100 µl of the conjugal mixture was spread on LB agar (Roth; Karlsruhe, Germany) containing Rif (50 mg liter^−1^), Kan (50 mg liter^−1^), CTX (2 mg liter^−1^), and TET (15 mg liter^−1^), and incubated at 37°C for 24 to 48 h. The transconjugants were verified by BOX-PCR and further tested for the antibiotic resistance phenotypes and genotypes as described above.

### Plasmid DNA extraction and detection by Southern blot hybridization.

Plasmid DNA extraction from TET-resistant transconjugants (pBC1.1, pBC1.3, pBC1.9, and pBC1.12) captured exogenously from the enrichment of cilantro leaves was performed using Qiagen Plasmid Mini Kit (Qiagen, Inc., Hilden, Germany) according to the manufacturer’s instructions. In order to detect the IncP-1 plasmids with Southern blot hybridization in these transconjugants, plasmid DNA was digested with the restriction enzyme NotI (Thermo Fisher Scientific, Waltham, MA, USA), and fragments were separated by electrophoresis on a 1% agarose-TBE gel as described previously (72). Southern blot hybridization was performed with digoxigenin (DIG)-labeled probes generated from PCR amplicons which were obtained from reference plasmid R751 for IncP1-β as previously described by Binh et al. (73).

### Plasmid replicon typing.

PBRT was used to identify the incompatibility group of plasmids in TET-resistant E. coli and transconjugants, and to confirm the presence of IncF and IncI plasmids as determined via the newly developed RT-PCR method as described above. This was done by PCR amplification on genomic DNA of the strains using primer sets for 30 replicons, HI1, HI2, I1, I2, X1, X2, X3, X4, L, M, N, FIA, FIB, FIC, FII, FIIS, FIIK, FIB KN, FIB KQ, W, Y, P1, A/C, T, K, U, R, B/O, HIB-M, and FIB-M, representative of major plasmid incompatibility groups among Enterobacteriaceae (74, 28). PCR products were separated by electrophoresis on a 2.5% agarose-TBE gel and stained with ethidium bromide.

### Detection of IncF and IncI plasmids, *tet*(A), and the class 1 integrase gene *intI1* via PCR-Southern blot hybridization and RT-PCR in TC-DNA.

PCR-Southern blot hybridization or RT-PCR was used to detect *tet*(A), *intI1*, IncF, and IncI plasmids in TC-DNA extracted from the microbial fraction detached from leaves directly or after an enrichment step on days 0 and 7. The PCR products were separated on a 1% agarose-TBE gel electrophoresis and then transferred to a positively charged nylon membrane (GE Healthcare, UK). Southern blot hybridization was carried out with digoxigenin (DIG)-labeled probes generated from PCR amplicons, which were obtained from reference plasmids pKJK5 for *intI1* and *tet*(A), as described by Dealtry et al. (75), R64 for IncI1, and pHNSHP45 for IncI2 and IncF. The primers and PCR conditions are listed in Table 4.
